# Weighted protein residue networks based on joint recurrences between residues

**DOI:** 10.1186/s12859-015-0621-1

**Published:** 2015-05-26

**Authors:** Wael I. Karain, Nael I. Qaraeen

**Affiliations:** 1 Department of Physics, Birzeit University, Birzeit, Palestine,; 2 Department of Computer Science, Birzeit University, Birzeit, Palestine,

**Keywords:** Joint Recurrence, Protein Residue Networks, Solvent Dynamics, Molecular Dynamics

## Abstract

**Background:**

Weighted and un-weighted protein residue networks can predict key functional residues in proteins based on the closeness centrality *C* and betweenness centrality *B* values for each residue. A static snapshot of the protein structure, and a cutoff distance, are used to define edges between the network nodes. In this work we apply the weighted network approach to study the β-Lactamase Inhibitory Protein (BLIP). Joint recurrences extracted from molecular dynamics MD trajectory positions of the protein residue carbon alpha atoms are used to define edge weights between nodes, and no cutoff distance is used. The results for *B* and *C* from our approach are compared with those extracted from an un-weighted network, and a weighted network that uses interatomic contacts to define edge weights between nodes, respectively.

**Results:**

The joint recurrence weighted network approach performs well in pointing out key protein residues. Furthermore, it seems to emphasize residues with medium to high relative solvent accessibility that lie in loop regions between secondary structure elements of the protein.

**Conclusions:**

Protein residue networks that use joint recurrences extracted from molecular dynamics simulations of a solvated protein perform well in pointing to hotspot residues and hotspot clusters. This approach uses no distance cutoff threshold, and does not exclude any interactions between the residues, including water-mediated interactions.

## Background

The network paradigm has been used extensively to investigate protein structure and function [1-6]. The nodes in the network represent the protein residues. The edges between the nodes represent the strength of the residue interactions. The importance of a node can be predicted by calculating two parameters. The first is the closeness centrality *C* which is defined as

1 $$ {C}_n=\frac{j-1}{{\displaystyle \sum_{i\ne n}sd\left(i,n\right)}} $$

where *sd* (*i*, *n*) is the shortest path between nodes *i* and *n*, and *j* is the number of nodes in the network. A node with a high *C* plays a principal part in the transmission of information to all other residues in the network [7]. The second is the betweenness centrality *B* which ranks the nodes according to the number of shortest paths passing through them between all the node pairs in the network. A node with a large *B* value controls the flow of information in a network [8]. Nodes with large *B* and *C* values have been shown to lie in critical regions in proteins, and are usually binding free energy hotspots, or are located in the vicinity of hotspots [9, 2, 10]. Protein hotspot residues play a key role in protein-protein interactions. They can be detected experimentally using alanine mutagenesis [11].

Weighted and un-weighted protein residue interaction networks are based mostly on a static protein structure. The network nodes represent either the carbon alpha or beta atoms. In un-weighted networks, an edge of weight 1 is defined between nodes that are less than a cutoff distance apart. This distance is usually taken between 4 and 8.5 Å. In weighted networks, edge weights are based on inter-residue interaction strengths, which in turn depend on cutoff threshold distance. A typical example is a graph spectral method which was used to find central residues in proteins [5, 12, 13]. Edge weights were set equal to the reciprocal of the inter-node distance, given that the two residues satisfied a minimum interaction strength threshold subject to a distance cutoff threshold. To improve predictions, edge weights were also based on snapshots of the protein structure provided by calculation intensive molecular dynamics MD simulations. For example, two residues within a certain cutoff distance from each other in a certain percentage of MD trajectory frames were considered to be connected. Linear and nonlinear correlation coefficients between residues within a certain threshold cutoff distance, were also used to define edge weights [14, 15]. Average values of non-bonded energy interactions between residues incorporated the chemical nature of residue interactions [16]. However, these methods ignored any long range residue-residue interactions, especially those by the surrounding water solvent [17]. This crucial coupling, facilitated by the forming and breaking of hydrogen bonds in the hydrogen bond network at the interface between the protein and the solvent, plays an important part in the dynamics of proteins [18-26]. It was taken into consideration by Bhattacharyya *et al*. who used an implicit solvent model to account for the effect of water on non-covalent energy interactions between two residues in a weighted protein residue interaction network [27]. This approach was successfully used to probe the allosteric mechanism in Pyrrolysyl-tRNA Synthetase. As a further refinement, we propose a method whereby edge weights can be defined in a way that includes all interactions, regardless of their nature, range, or value.

We have recently used the method of correlation of probability of recurrence CPR to find linear and nonlinear correlation coefficients between protein residue carbon alpha atoms [28]. This technique is based on the recurrence plot RP method [29]. Recurrence plots were introduced to visualize high dimensional phase space trajectories of dynamical systems using a 2-D map of the system’s recurrences. The phase space trajectory of a given dynamical system is reconstructed using the method of time delays [30, 31]. Consequently, the recurrence plot locates recurring patterns in the dynamics of a system without making any previous assumptions about the nature of the dynamics. This technique can be especially useful when dealing with a-periodic and non-stationary systems, such as complex biological systems. To avoid the human bias inherent in studying graphical maps, the RP method was later developed to give quantitative results, and became known as recurrence quantification analysis RQA [32]. It has been used in many fields, including protein structure and dynamics [33]. The simultaneous recurrences by two or more systems, can also be detected using a multivariate version of the recurrence plot [34]. This joint recurrence plot JRP method can be used to quantify synchronization between two or more coupled dynamical systems. We hypothesize that if two residue carbon alpha atoms recur to their corresponding previous states at the same time instant, then they are interacting at that time instant. There is no need to know the nature of the interaction. By summing the total number of simultaneous recurrences over a given time period, an edge weight can be defined between the two atoms. A residue pair with a large number of joint recurrences is assumed to interact strongly. No threshold cutoff distance is needed to define the range of the interaction. However, each edge weight is divided by the geometric distance between the two residues [5]. This is justified because geometrically close residues with a given number of joint recurrences are expected to interact stronger than two residues that are far apart with the same number of joint recurrences. All interaction mechanisms, including those mediated by the solvent, are thus included.

The protein system we study in this work is the 165 residue β-Lactamase Inhibitory Protein BLIP. It is secreted by the soil bacterium *Streptomyces clavuligerus*. It inhibits β-lactam enzymes, which hydrolyze β-lactam antibiotics and nullify their effect [35-37]. It has been investigated extensively as a model system to understand the principles of affinity and specificity in protein-protein interactions [38]. It has a large (2636 Å^2^), concave, solvent exposed interaction interface consisting of an eight-stranded β-sheet. Numerous mutagenesis studies have pinpointed the hotspot residues that dominate its binding free energy with a number of β-Lactamase enzymes. These key residues are now known to be located in six independent hotspot clusters: C1:49; C2:74,142,143; C3:148,150,160; C4: 112,162; C5:36, 41, 50, 53; C6: 71,113 [39-45]. Most notably, residues Asp 49(C1) and Phe 142(C2) play a critical role in the binding process [37, 39]. Residue 49(C1) also plays a dominant role in information flow between some of the hotspot clusters [46]. An ammonium ion binding site lies at residues 6 and 7 of BLIP. The sulfate ion binding site is at residues 12, 13, 14, and 70. This protein is suitable for our work on solvent-protein coupled dynamics due to the expected role the solvent pslay at such a large, extensively hydrated, interaction surface [47, 48].

In this work, a weighted network will be prepared for the BLIP protein. The residue carbon alpha atoms will be the nodes. Edge weights will be given by the cumulative number of joint recurrences of a residue pair over a time period of 10 ns, divided by the distance between the two carbon atoms. The closeness and betweenness centrality values for each node will be calculated. For comparison, the BLIP protein will also be represented as an un-weighted network with a cutoff threshold distance of 7 Å, and a weighted network with edge weights based on inter-atomic contacts, calculated by the CSU program [49]. The results from these three networks will be compared.

## Results and discussion

**Fig. 1:**
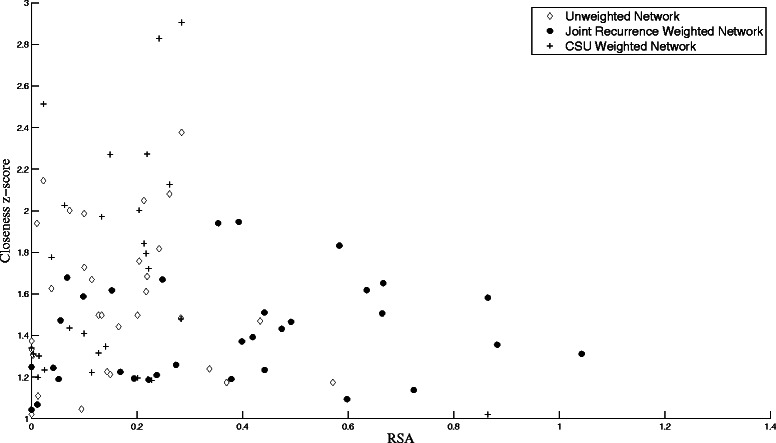
The closeness centrality *C* versus the relative solvent accessibility RSA. This figure shows the *C* z-scores larger than 1 for the BLIP residues versus their RSA values for the un-weighted network with a 7 Å cutoff threshold (◊), the CSU weighted network (+), and the JRP weighted network (●)

**Fig. 2:**
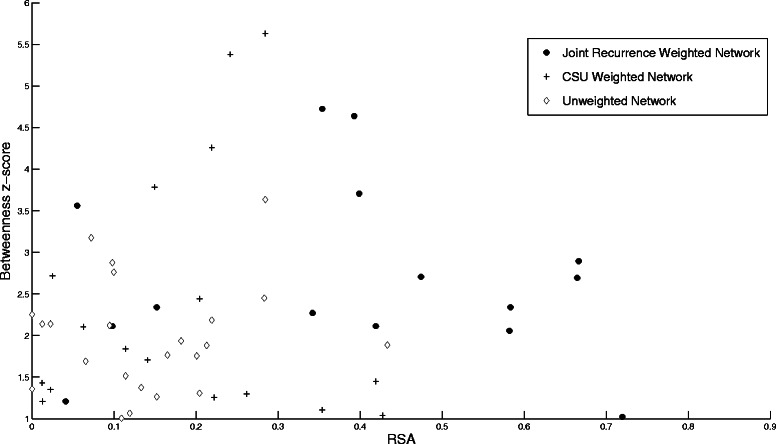
The betweenness centrality *B* versus RSA. This figure shows the *B* z-scores larger than 1 for the BLIP residues versus their RSA values for the un-weighted network with a 7 Å cutoff threshold (◊), the CSU weighted network (+), and the JRP weighted network (●)

**Fig. 3:**
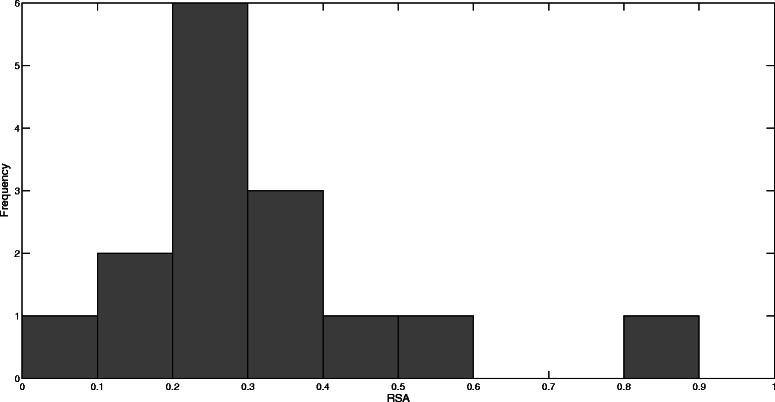
The relative solvent accessibility RSA histogram for the BLIP hotspot residues. This figure shows the RSA value distribution for the experimentally determined hotspot residues in BLIP

We start by examining the solvent accessibility for residues with significant *B* and *C* values from the three networks for BLIP. Fig 1 shows a scatter plot of the *C* z-score values versus the corresponding residue relative solvent accessibility RSA values. A significance z-score value of 1 is chosen arbitrarily to give a representative sample for each network. The average RSA values are 0.17, 0.17, and 0.37, for the un-weighted, CSU-weighted, and JRP-weighted networks, respectively. Similarly, Fig. 2 shows a scatter plot of the *B* z-score values larger than 1 versus the corresponding residue RSA values for the three networks. The average RSA values are 0.15, 0.18, and 0.40, for the un-weighted, CSU-weighted, and JRP-weighted networks, respectively. The significant residues extracted from the JRP-weighted network have mostly medium and high RSA values. Amitai *et al*. [9] reported that the optimal parameters for protein active site prediction for a set consisting of 178 protein chains of enzymes with a total of 567 active sites were *C* values ≥ 1.1, and RSA values of 4.5–40 %. However, they also reported a general tendency for residues with high *C* to be unexposed. Del Sol *et al*. [50] considered only residues with RSA < 20 % in their work to identify key residues. To see if this is justified in the case of BLIP, a histogram of the RSA values for the experimentally determined BLIP hotspot residues is shown in Fig. 3. The majority of these residues has RSA values larger than 20 %, and can thus be regarded as being at least partially exposed [9]. In fact, the average RSA value for these hotspot residues is 31 %, ranging from 3 to 86 %. Thus, even though the conventional wisdom is to narrow the search for key residues among buried or partially exposed residues, one should not generalize this approach. Solvent exposed residues should be expected to play an important part in the control and flow of information inside the protein network.

**Fig. 4:**
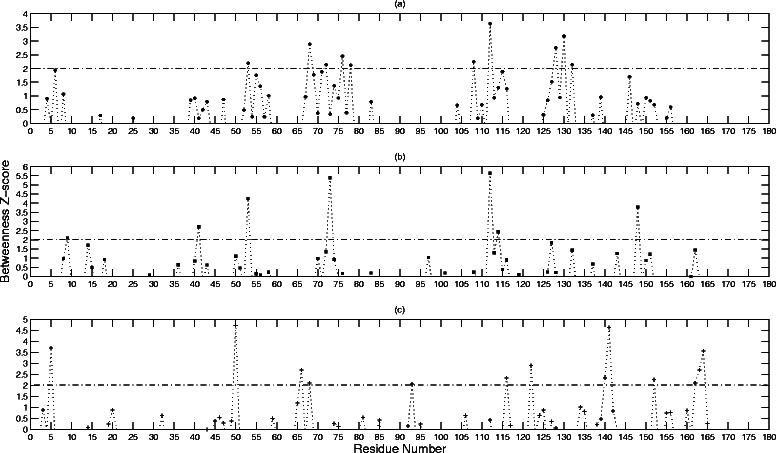
The standardized betweenness centrality *B* values for BLIP residues. This figure shows the standardized *B* values for the BLIP residues calculated from (**a**) un-weighted network, (**b**) CSU weighted network, and (**c**) JRP weighted network

**Fig. 5:**
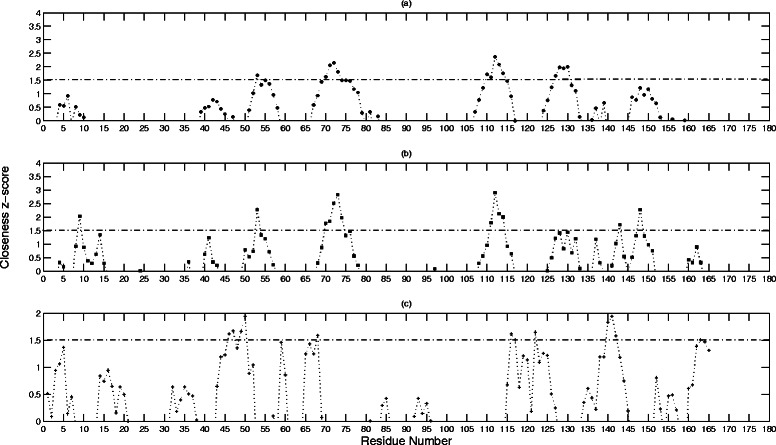
The standardized closeness centrality *C* values for BLIP residues. This figure shows the standardized *C* values for BLIP residues calculated from (**a**) un-weighted network, (**b**) CSU weighted network, and (**c**) JRP weighted network

Figures 4 and 5 show the standardized *B* and *C* values, respectively, for the residues represented as nodes in each of the three networks. The results are summarized in Tables 1, 2, and 3, for the un-weighted, CSU-weighted, and JRP-weighted networks, respectively. A z-score cutoff threshold of 2 is chosen arbitrarily for the *B* values, and 1.5 for the *C* values, respectively, to get a representative significant sample. Each table lists the significant nodes, and points out which ones are experimentally determined hotspots or ion binding sites. It also lists the first degree neighbors for each node, and points out if they are hotspot residues or ion binding sites as well.

**Table 1:** Betweenness centrality and closeness centrality results for significant nodes in the un-weighted network

Centrality	Residue #	RSA	Significant neighbors
Betweenness	53(C5)	0.22	41(C5), 112(C4)
-	68	0.10	Sulfate ion binding site
-	72	0.02	53(C5), 71(C6), 74(C2),112(C4)
-	76	0.03	Ammonium ion binding site, 74(C2)
-	78	0.09	-
-	108	0.00	-
-	112(C4)	0.28	53(C5), 71(C6), 148(C3)
-	128	0.10	112(C4), 150(C3)
-	130	0.07	112(C4), 148(C3)
-	132	0.01	-
Closeness	53(C5)	0.22	41(C5), 112(C4)
-	55	0.20	36(C5), 41(C5), 53(C5),71(C6), Sulfate ion binding site
-	70 (Sulfate ion binding site)	0.04	Sulfate ion binding site
-	71(C6)	0.21	53(C5), Sulfate ion binding site, 112(C4), 113(C6)
-	72	0.02	53(C5), 71(C6), 74(C2), 112(C4)
-	73	0.24	53(C5), 74(C2), 112(C4), 148(C3)
-	74(C2)	0.13	143(C2)
-	75	0.13	53(C5), 74(C2), Ammonium ion binding site
-	110	0.10	-
-	111	0.22	112(C4), Sulfate ion binding site
-	112(C4)	0.28	53(C5),71(C6),148(C3)
-	113(C6)	0.26	112(C4), 71(C6), Sulfate ion binding site
-	114	0.20	Sulfate ion binding site
-	127	0.11	150(C3)
-	128	0.10	112(C4), 150(C3)
-	129	0.01	112(C4), 148(C3)
-	130	0.07	112(C4), 143(C2), 148(C3)

This table lists residues with betweenness centrality z-score values larger than 2 and closeness centrality values larger than 1.5. It also gives the relative solvent accessibility for each residue, and its first degree neighbors. The symbols between brackets denote hotspot clusters

**Table 2:** Betweenness centrality and closeness centrality results for significant nodes in the CSU-weighted network

Centrality	Residue	RSA	Significant neighbors
Betweenness	9	0.06	Ammonium and Sulfate ion binding site, 71(C6)
-	41(C5)	0.03	36(C5), 53(C5)
-	53(C5)	0.22	41(C5), 112(C4)
-	73	0.24	53(C5), 74(C2), 112(C4), 143(C2)
-	112(C4)	0.28	53(C5), 71(C6), 148(C3)
-	114	0.20	Sulfate ion binding site
-	148(C3)	0.15	112(C4), 143(C2), 150(C3), 160(C3), 162(C4)
Closeness	9	0.06	71(C6), Ammonium and Sulfate ion binding sites
-	53(C5)	0.22	41(C5), 112(C4)
-	70 (Sulfate ion binding site)	0.04	112(C4), 71(C6), Sulfate ion binding site
-	71(C6)	0.22	53(C5),112(C4), 113(C6), Sulfate ion binding site
-	72	0.02	53(C5), 71(C6), 74(C2), 112(C4)
-	73	0.24	53(C5), 74(C2), 112(C4), 148(C3)
-	74(C2)	0.13	143(C2)
-	111	0.22	112(C4), Sulfate ion binding site
-	112(C4)	0.28	53(C5), 71(C6), 148(C3)
-	113(C6)	0.26	112(C4), 71(C6), sulfate ion binding site
-	114	0.20	Sulfate ion binding site
-	143(C2)	0.22	74(C2), 142(C2), 148(C3)
-	148(C3)	0.15	112(C4),143(C3), 150(C3), 160(C3)

This table lists residues with betweenness centrality z-score values larger than 2 and closeness centrality values larger than 1.5. It also gives the relative solvent accessibility for each residue, and its first degree neighbors. The symbols between brackets denote hotspot clusters

**Table 3:** Betweenness centrality and closeness centrality results for significant nodes in the JRP-weighted network

Centrality	Residue	RSA	Significant neighbors
Betweenness	5	0 .40	Ammonium ion binding site
-	50(C5)	0.35	36(C5), 41(C5), 49(C1)
-	66	0.47	-
-	68	0.10	Sulfate ion binding site
-	93	0.58	-
-	116	0.15	Sulfate ion binding site
-	122	0.67	-
-	140	0.58	74(C2)
-	141	0.39	74(C2), 142(C2), 143(C2)
-	152	0.34	-
-	162(C4)	0.42	148(C3), 150(C3), 160(C3)
-	163	0.67	142(C2)
-	164	0.05	-
Closeness	46	0.64	50(C5)
-	47	0.07	49(C1), 50(C5), 74(C2)
-	49(C1)	0.25	50(C5)
-	50(C5)	0.35	36(C5), 41(C5), 49(C1), 53(C5)
-	68	0.10	Sulfate ion binding site
-	116	0.15	Sulfate ion binding site
-	117	0.44	-
-	122	0.67	-
	140	0.58	74(C2)
	141	0.39	74(C2), 142(C2), 143(C2)
	142(C2)	0.86	143(C2)
	163	0.67	142(C2), 162(C4)

This table lists residues with betweenness centrality z-score values larger than 2 and closeness centrality values larger than 1.5. It also gives the relative solvent accessibility for each residue, and its first degree neighbors. The symbols between brackets denote hotspot clusters

**Fig. 6:**
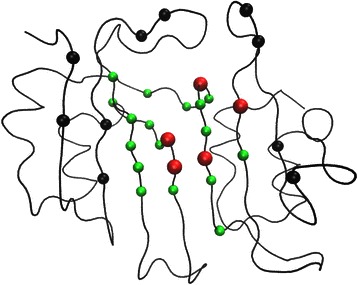
The locations of the significant un-weighted network residues inside BLIP. This figure shows the locations of significant BLIP residues with *B* z-score values larger than 2, and *C* z-score values larger than 1.5. The black colored spheres are experimentally determined hotspots. The red colored spheres are experimentally determined hotspots detected by this network. The green colored spheres are significant residues determined by this network

Table 1 lists residues with significant *B* and *C* values detected by the un-weighted network. This list contains five experimentally determined hotspot residues: 53(C5), 71(C6), 74(C2), 112(C4), and 113(C6). The RSA values for these hotspots range from 0.13 to 0.28. Five hotspot clusters, as well as the sulfate ion and the ammonium ion binding sites, are among the first neighbors for residues in this list. The only cluster missing is C1, which consists of the key residue 49. Fig 6 shows the locations of these residues inside BLIP. In general, they are situated in the middle of the concave shaped active site.

**Fig. 7:**
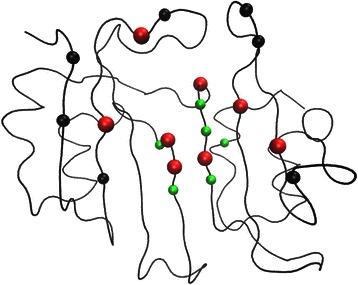
The locations of the significant CSU-weighted network residues inside BLIP. This figure shows the locations of significant BLIP residues with *B* z-score values larger than 2, and *C* z-score values larger than 1.5. The black colored spheres are experimentally determined hotspots. The red colored spheres are experimentally determined hotspots detected by this network. The green colored spheres are significant residues determined by this network

Table 2 lists significant residues detected by the CSU-weighted network. This list contains eight experimentally determined hotspot residues: 41(C5), 53(C5), 71(C6), 74(C2), 112(C4), 113(C6), 143(C2), and 148(C3). The RSA values for these hotspot residues range between 0.03 and 0.28. Five hotspot clusters, as well as the sulfate ion and the ammonium ion binding sites, are among the first neighbors for the residues in this list. Cluster C1 is again not detected. Fig 7 shows the locations of these residues in BLIP. They are situated at the edge and the center of the active site.

**Fig. 8:**
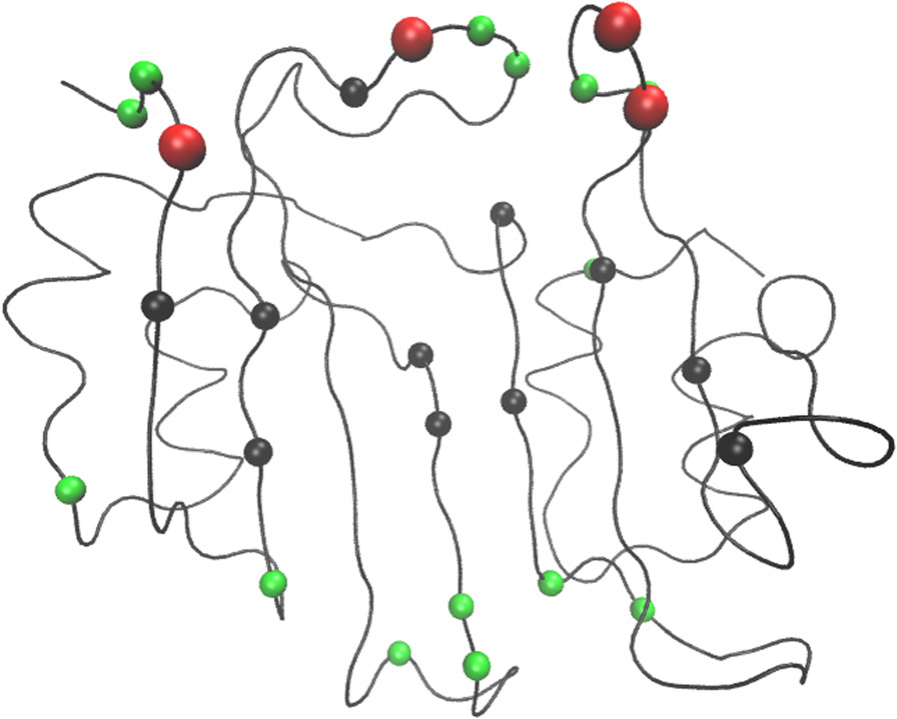
The locations of the significant JRP-weighted network residues inside BLIP. This figure shows the locations of significant BLIP residues with *B* z-score values larger than 2, and *C* z-score values larger than 1.5. The black colored spheres are experimentally determined hotspots. The red colored spheres are experimentally determined hotspots detected by this network. The green colored spheres are significant residues determined by this network

Table 3 lists significant residues in the JRP-weighted network. This list contains four experimentally determined hotspot residues: 49(C1), 50(C5), 142(C2), and 162(C4). The RSA values for these hotspot residues range from 0.25 to 0.86. Five hotspot clusters, including C1(49), as well as the sulfate ion and the ammonium ion binding sites, are among the first neighbors for the residues in this list. Only cluster C6 (71,113) is not detected. Most notably, the two “anchor” hotspot residues 49(C1) and 142(C2) are detected by the JRP-weighted network [51], while the two other networks fail to detect these important residues. Fig 8 shows the locations of these residues in BLIP. In clear contrast to the results in the un-weighted and CSU-weighted networks, these residues are located at the periphery of the protein interface surface.

Five BLIP residues are regarded as consensus hotspots for binding with the TEM-1, SHV-1, SME-1, and Bla1 enzymes: 36, 41, 49, 53, and 150. The residues 148, 160, and 162 are hotspots when BLIP binds all of the above enzymes with the exception of SHV-1 [40, 41]. The residues 73, 74, and 50, are considered binding specificity determinants [52,53]. The un-weighted network detects residue 53(C5, RSA = 0.22) from the first group, and residue 74(C2, RSA = 0.13) from the third group. It does not detect any of the residues from the second group. The CSU-weighted network detects residues 41(C5, RSA = 0.03) and 53(C5, RSA = 0.22) from the first group, residue 148(C3, RSA = 0.15) from the second group, and residue 74(C2, RSA = 0.13) from the third group. The JRP-weighted network detects residue 49(C1, RSA = 0.25) from the first group, residue 162(C4, RSA = 0.42) from the second group, and residue 50(C5, 0.35) from the third group. Again, the hotspots detected by the JRP-weighted network tend to have higher RSA values. This should be noteworthy since the significant hydration of the BLIP interface surface, has been proposed as a major factor in its “promiscuity”, and ability to bind multiple enzymes [37,48]. It is also worth noting that a significant number of the residues with large *B* and *C* values extracted from the JRP weighted network, lie in the flexible loop regions between the secondary structure elements of the protein. It is also interesting that even though the hotspot residue 49 has a relatively low RSA value of 0.25, it was not detected directly by the un-weighted or the CSU-weighted networks.

## Conclusions

In this work we introduce the use of joint recurrences to represent interaction strengths between residue pairs in a weighted protein residue network. We also remove the cutoff distance condition used in defining edges, thus keeping long range interactions. We use betweenness and closeness centralities to detect key hotspot residues in the BLIP protein, which has a large, solvent exposed interaction surface. Our JRP-weighted network results compare well with those extracted from an un-weighted network with a 7 Å cutoff distance, and a weighted network with the edge weights defined by the number of the inter-atomic contacts between residues. While the CSU-weighted network detected more hotspots, the JRP-weighted network was able to detect the “anchor” residues 49(C1) and 142(C2). Our results indicate that residues with significant *B* and *C* values are either themselves, or are in contact with, hotspot residues or ion binding sites. Our approach also favors residues that lie in loop regions between secondary structure elements, and that are partially or highly exposed to the surrounding solvent. This seems more appropriate for this “promiscuous” protein, which has experimentally determined hotspot residues that tend to be either partially or highly exposed to the solvent, and which presents a large interaction surface to the solvent. In general, protein networks should not neglect any interaction mechanisms.

## Methods

The starting point in reconstructing the phase space trajectory *X* (*t*) of a given dynamical system, is a scalar time series of one of the system’s measured or calculated observables *Z*(*t*), where *Z = (Z*_*1*_ *,Z*_*2*_ *,….,Z*_*N*_ *)* consists of evenly spaced single values 1 to N. The reconstructed trajectory reflects the underlying dynamics of the original system. Each point in the reconstructed phase trajectory represents a possible state for that system, and is given by a vector

2 $$ \overrightarrow{X_i}=\left({Z}_i,{Z}_{i+d},....,{Z}_{i+\left(k-1\right)d}\right)\kern0.72em k=1,\dots, m $$

where *d* is the delay parameter, and *m* is the embedding dimension. The delay parameter *d* is estimated by finding the first minimum in the mutual information function. This function can detect linear and nonlinear correlations between the elements of the scalar time series [54]. A suitable delay parameter should give the least amount of shared information between the vector components, while at the same time keeping them related. The embedding dimension *m* is then estimated using the method of false nearest neighbors [55]. Choosing the correct embedding dimension helps to “unfold” the trajectory. For example, if one inspects a circular orbit along its edge, two points that are not true neighbors could appear to be neighbors due to the low embedding dimension. By using a higher dimension to inspect this circle, in this case two, one can differentiate between true and false neighbors. The embedding dimension chosen would be where the number of false nearest neighbors is very small. The RP then shows the recurrence of a state *X*_*i*_ at time *i* to a former state *X*_*j*_ at time *j* in the phase space trajectory if these two states are within a threshold norm distance ε from each other. The mathematical expression of the RP matrix is:

3 $$ {\displaystyle {R}_{i,j}}\left(\varepsilon \right)=\varTheta \left(\varepsilon -\left\Vert {\displaystyle {X}_i}\right.\right.-\left.\left.{\displaystyle {X}_j}\right\Vert \right)\kern2.4em i,j=1,..........,N $$

where *N* is the number of states, ε is a threshold distance, Θ is the Heaviside function (Θ(x) = 0 if *x* < 0 and 1 otherwise), and ∥.∥ is the Euclidean norm. A recurrence (*R*_*ij*_ = 1) in the matrix is represented as a black dot in the plot. The rate of the number of black dots to the total number of points in the matrix gives the recurrence rate value. The Euclidean norm is used due to its intermediate value of neighbors between the maximum and minimum norms. The threshold parameter ε is the limit that transforms the distance matrix between the states into a recurrence plot of 1’s and 0’s [33]. A too small ε would give a small number of recurrences, while a too large ε would give too many recurrences. This threshold can be defined using different methods [33]. In this work, we chose ε to give a recurrence rate of 3 %. If one is interested in studying how two or more dynamical systems are related to each other, joint recurrences JRP can be used [34], as defined by

4 $$ {\displaystyle {JR}_{i,j}^{x,y}}\left({\displaystyle {\varepsilon}^x}{\displaystyle {\varepsilon}^y}\right)=\varTheta \left({\displaystyle {\varepsilon}^x}-\left\Vert {\displaystyle {X}_i}\right.\right.-\left.\left.{\displaystyle {X}_j}\right\Vert \right)\ \varTheta \left({\displaystyle {\varepsilon}^y}-\left\Vert {\displaystyle {Y}_i}\right.\right.-\left.\left.{\displaystyle {Y}_j}\right\Vert \right), \kern1.59em i,\ j=1,\dots N $$

where the JRP has a value of one at time instants when the two systems recur to their simultaneous previous states, and zero otherwise. Equation 4 can be generalized to as many dynamical systems as is needed.

The molecular dynamics simulation and related analysis in this work are performed using the molecular dynamics computer programs NAMD [56] and VMD [57]. The starting BLIP protein structure is downloaded from the protein data bank (PDB: 3gmu) [45]. The simulation details are described elsewhere in detail [28]. The phase space trajectory for each protein residue carbon alpha atom is constructed from a scalar time series of the root mean square deviation RMSD of its position over time [28]. The RMSD values are calculated after removing translational and rotational atomic motions using least square fitting. Each time series consists of 1000 points evenly separated by 10 ps, for a total time of 10 ns for each series. The recurrence parameters of embedding dimension and delay are calculated using the VRA program [58]. The threshold parameter is set to give a recurrence rate of 3 %. The recurrence matrices are calculated for all 165 residue carbon alpha atoms using the CRP toolbox routines [59]. By inspecting the recurrence matrices for all 165 residue carbon alpha atoms at all time instants, the number of joint recurrences for each residue pair are accumulated over 10 ns. This number of joint recurrences constitutes the edge weight between the two residues. Each edge weight is consequently divided by the geometric distance between the corresponding residue carbon alpha atoms [6]. For comparison, an un-weighted network with a cutoff threshold distance of 7 Å is prepared. Similarly, a weighted network is prepared with edge weights based on inter-atomic contacts calculated by the CSU program [49]. The *B* and *C* values for the three networks are then calculated using the MatlabBGL network toolbox [60]. These values are then standardized by subtracting the mean value, and dividing by the standard deviation for each distribution, respectively. Thus the *B* and *C* values for each residue are given as z-scores.

The degree of exposure of each residue to the surrounding water solvent is given by the RSA value for that residue. The RSA values are calculated by dividing the residue’s accessible surface area ASA as given by the CSU program [49], by the maximum solvent-accessible surface area of the corresponding standard amino acid residue as calculated recently [61]. Residue with RSA <5 %, RSA ≥ 5 % and < 20 %, and RSA ≥20 %, are considered buried, partially buried, and exposed, respectively [50].

## Abbreviations

ASA: Accessible surface area
B: betweenness centrality
Bla1: β-lactamase protein
BLIP: β-Lactamase Inhibitory Protein
C: Closeness centrality
CPR: Correlation of probability of recurrence
CSU: Contact of structural units
JRP: Joint recurrence plot
NAMD: Not another molecular dynamics
PDB: Protein data bank
RMSD: Root mean square deviation
RP: Recurrence plot
RQA: Recurrence quantification analysis
RSA: Relative solvent accessibility
SHV-1: SME-1, TEM-1: β-lactamase proteins
VMD: Visual molecular dynamics
VRA: Visual recurrence analysis
